# Adverse Event Profiles of PARP Inhibitors: Analysis of Spontaneous Reports Submitted to FAERS

**DOI:** 10.3389/fphar.2022.851246

**Published:** 2022-03-25

**Authors:** Xiaojiang Tian, Lin Chen, Di Gai, Sijie He, Xuan Jiang, Ni Zhang

**Affiliations:** ^1^ Department of Pharmacy, Chongqing Health Center for Women and Children, Chongqing, China; ^2^ Department of Pharmacy, Beijing Obstetrics and Gynecology Hospital, Capital Medical University, Beijing Maternal and Child Health Care Hospital, Beijing, China; ^3^ Department of Pharmacy, Maternal and Child Health Hospital of Hubei Province, Tongji Medical College, Huazhong University of Science and Technology, Wuhan, China; ^4^ Department of Oncology, The Second Affiliated Hospital of Chongqing Medical University, Chongqing, China

**Keywords:** PARP inhibitors, adverse events, FDA adverse events reporting system, reporting odds ratios, signal detection

## Abstract

**Background:** Several poly ADP ribose polymerase inhibitors (PARPis) are currently approved for the treatment of a variety of cancers. The safety profile of PARPis has not yet been systemically analyzed in the real world. We conducted this pharmacovigilance analysis using the US FDA’s Adverse Event Reporting System (FAERS) database to explore the difference in adverse events (AEs) among PARPis.

**Method**s: FAERS data (December 2014 to October 2021) were searched for reports of all FDA-approved PARPis across all indications. We used the standardized MedDRA query (SMQ) generalized search AEs on the preferred term (PT) level based on case reports. After filtering duplicate reports, disproportionality analysis was used to detect safety signals by calculating reporting odds ratios (ROR). Reports were considered statistically significant if the 95% confidence interval did not contain the null value.

**Results**: Within the standardized MedDRA queries, significant safety signals were found, including those for olaparib [blood premalignant disorders (ROR = 17.06)], rucaparib [taste and smell disorders (ROR = 9.17)], niraparib [hematopoietic throbocytopenia (ROR = 28.2)], and talazoparib [hematopoietic erythropenia (ROR = 9.38)]. For AEs on the PT level, we found several significant signals, including platelet count decreased with niraparib (ROR = 52.78); red blood cell count decreased with niraparib (ROR = 70.47) and rucaparib (ROR = 15.09); myelodysplastic syndrome with olaparib (ROR = 35.47); acute myeloid leukaemia with olaparib (ROR = 25.14); blood pressure fluctuation with niraparib (ROR = 20.54); lymphangioleiomyomatosis with niraparib (ROR = 471.20); photosensitivity reaction with niraparib (ROR = 21.77) and rucaparib (ROR = 18.92); renal impairment with rucaparib (ROR = 33.32); and interstitial lung disease with Olaparib (ROR = 11.31). All the detected safety signals were confirmed using signals of disproportionality reporting methods.

**Conclusion**: PARPis differed in their safety profile reports. The analysis of the FAERS database revealed significant safety signals that matched previously published case reports, including serious gastrointestinal, blood and lymphatic system, cardiovascular and respiratory complications, which require individualized drug administration according to patients’ conditions.

## Introduction

PARPis are a class of anticancer drugs that target the inhibition of poly ADP ribose polymerase (PARP), which can affect the self-replication of cancer cells. It was also the first targeted drug to successfully use the concept of synthetic lethality to obtain clinical approval. Under normal circumstances, a large number of DNA single-strand breaks produced by human cells can be repaired through the PARP-mediated base-excision repair (BER) pathway (Ashworth and Lord, 2018; Jain and Patel, 2019). PARP proteins are responsible for DNA damage detection and signal transduction. PARPis are able to interact with the binding site for the PARP cofactor (NAD+) and trap PARP on DNA. In this way, they inhibit single-strand DNA damage repair. In the case of homologous recombination deficiency (HRD), DNA double-strand breaks cannot be repaired. The two effects overlap and increase cell death (Satoh and Lindahl, 1992).

At present, many PARPis have been approved by the FDA, such as olaparib, niraparib, rucaparib and talazoparib, covering ovarian cancer, prostate cancer, pancreatic cancer, breast cancer and other cancer types (J. de Bono et al., 2020; J. S. de Bono et al., 2021; Poveda et al., 2021; Wu et al., 2021). With the widespread use of PARPis, drug-related adverse reactions(AEs) have also attracted much attention. In pivotal PARPi RCTs, the most common AEs that have led to dose modifications were hematologic toxicities; other common AEs associated with this class of therapy include gastrointestinal disorders, photosensitivity, and fatigue (Mirza et al., 2016; Pujade-Lauraine et al., 2017). A recent meta-analysis showed that niraparib and rucaparib had higher risks for hematological and gastrointestinal toxicities, and olaparib has a higher risk of serious adverse events (SAEs), such as myelodysplastic syndrome (MDS) and acute myeloid leukemia (AML) (Bao et al., 2021; Morice et al., 2021). Unfortunately, there is little knowledge about differences between PARPis.

Data-mining techniques, such as signal detection algorithms, are increasingly being used to explore medical databases and analyze large volumes of accumulated data to identify potential associations between drugs and AEs that may have escaped detection in clinical trials (Wilson et al., 2004). The FAERS is one of the largest databases of AEs designed to support the FDA postmarketing safety surveillance program for approved drugs and biologics. Data from FAERS are publicly available and routinely used by the FDA, health systems, clinical scientists, and pharmaceutical manufacturers to identify potential safety signals. The purpose of this study was to use data mining technology to detect and analyze the signals of AEs of representative PARPi after-market drugs, including blood toxicity, gastrointestinal toxicity and neurotoxicity, to explore the AEs of each drug in the real world.

## Methods

### Data Sources

The data for this study were retrieved from the public release of the FAERS database, which adheres to the international safety reporting guidance issued by the International Conference on Harmonisation (ICH E2B). OpenVigil FDA, a pharmacovigilance tool, was adapted to extract FAERS data. The classification and standardization of AEs in the FAERS data refer to the Medical Dictionary for Regulatory Activities (MedDRA). In the FAERS database, each report is coded using preferred terms (PTs) from the MedDRA terminology; a given PT can be assigned to one or more High-level Terms (HLT), High-level Group Terms (HLGT), and System Organ Class (SOC) levels in MedDRA. In addition, different PTs can be combined to define a specific clinical syndrome through an algorithmic approach known as standardized MedDRA queries (SMQs). This study relied on definitions used by MedDRA.

### Data Processing

From “December 2014” to “October 2021,” FAERS reports listing “olaparib,” “niraparib,” “rucaparib” and “talazoparib” as suspected (“primary suspect” or “secondary suspect”) were analyzed after removing duplicate reports (with the same ID number). Two researchers used SMQ and PT to classify PARPi-related AEs and extracted patient and drug information from reports. The signals of disproportionality reporting (SDR) were evaluated using the established pharmacovigilance index ROR (van Puijenbroek et al., 2002; Bate and Evans, 2009). Then, a two-by-two contingency table was constructed (Table 1), and disproportional AEs and drug combinations were identified. Cases were represented by AEs in which PARPi were mentioned by the reporter as suspected (“Primary Suspect” or “Secondary Suspect”); noncases were all other AEs. ROR values were calculated as (a × d)/(b × c) and expressed as point estimates with 95% confidence intervals (CIs).

**TABLE 1:** Two-by-two contingency table for disproportionality analyses.

	Adverse events of interest	All other adverse events of interest	Total
Drug of interest	a	b	a+b
All other drugs of interest	c	d	c + d
Total	a+c	b + d	a+b + c + d

Reporting odds ratio (ROR) = (a/c)/(b/d) = ad/bc; 95% CI, e^ln (ROR) ±1.96√(1/a+1/b+1/c+1/d)^

### AEs Signal Detection

Descriptive analysis was used to analyze the AEs in relation to olaparib, niraparib, rucaparib and talazoparib in the FAERS database. The ROR signal detection method was used to detect the signal strength. The larger the ROR value, the stronger the signal. SPSS 23.0 was used for statistical analysis. The count data were expressed as frequency (%), and the comparison of count data between groups was conducted by χ2 test.

## Results

### Descriptive Analysis

As of October 2021, a total of 13,703,970 AE reports were submitted to FAERS, including 6,863 reports for olaparib, 6,382 reports for rucaparib, 10,361 reports for niraparib and 535 reports for talazoparib. Table 2 describes the characteristics of AE reports submitted for PARPi. The patient gender in the reports was mainly female, including 77.68% for olaparib, 92.21% for rucaparib, 76.05% for niraparib and 61.50% for talazoparib. The age of the patients was mainly ≥65 years old (65.16% for olaparib, 82.48% for rucaparib, 49.41% for niraparib and 82.80% for talazoparib). The main outcome of olaparib AEs was death (*n* = 2,216), but that of the other three PARPi was hospitalization (rucaparib *n* = 1,074; niraparib *n* = 2,612; talazoparib *n* = 209). Table 3 lists the high-frequency indications and concomitant medications (top three) associated with the use of olaparib, rucaparib, niraparib and talazoparib in the AE reports.

**TABLE 2:** Clinical characteristics of patients with PARPi AEs from the FAERS database.

Characteristics	Reports, N (%)			
	Olaparib	Rucaparib	Niraparib	Talazoparib
Gender				
Male	926(13.49)	252(3.95)	219(2.11)	125(23.36)
Female	5,331(77.68)	5,885(92.21)	7,880(76.05)	329(61.50)
Unknown	606(8.83)	245(3.84)	2,262(21.84)	81(15.14)
Age				
<18	7(0.01)	5(0.08)	10(0.10)	2(0.37)
18–64	1713(24.96)	682(10.69)	3,850(37.16)	81(15.15)
≥65	4,472(65.16)	5,264(82.48)	5,119(49.41)	443(82.80)
Unknown	671(9.78)	431(6.75)	1,392(13.43)	9(1.68)
Reporter				
Physician	2,333(33.99)	569(8.92)	1,314(12.68)	215(40.19)
Pharmacist	258(3.76)	54(0.85)	107(1.03)	35(6.54)
Other Health Professional	2,788(40.72)	4,059(63.60)	576(5.56)	165(30.84)
Consumer	1,350(19.67)	1,697(26.59)	8,110(78.27)	95(17.76)
Lawyer	1(0.01)	0(0.00)	2(0.02)	0(0.00)
Unknown	134(1.95)	3(0.05)	252(2.43)	25(4.67)
Outcome of AEs				
Hospitalization (initial or prolonged)	1,238(18.03)	1,074(16.83)	2,612(25.21)	209(39.07)
Disability	81(1.18)	10(0.16)	32(0.31)	2(0.37)
Life-threatening	256(3.73)	45(0.71)	598(5.77)	30(5.61)
Death	2,216(32.29)	325(5.09)	762(7.35)	122(22.80)
Other	3,072(44.76)	49.28(77.22)	5,243(61.36)	172(32.15)

**TABLE 3:** Top three indications and concomitant medications for PARPi AEs from the FAERS database.

	Olaparib (N)	Rucaparib (N)	Niraparib (N)	Talazoparib (N)
Indications	Ovarian Cancer (3,091)	Ovarian Cancer (5,115)	Ovarian Cancer (7,529)	Breast Cancer (227)
	Prostate Cancer (267)	FallopianTube Cancer (278)	FallopianTube Cancer (659)	Prostate Cancer (183)
	Breast Cancer(479)	Malignant Peritoneal Neoplasm (263)	Malignant Peritoneal Neoplasm (263)	Ovarian Cancer (9)
Concomitant Medications	Carboplatin (310)	Ondansetron (527)	Ondansetron (777)	Enzalutamide (59)
	Bevacizumab (271)	Lorazepam (225)	Ergocalciferol (471)	Avelumab (49)
	Paclitaxel (253)	Gabapentin (224)	Lorazepam (412)	Ondansetron (43)

### Signal of Standardized MedDRA Queries

**FIGURE 1:**
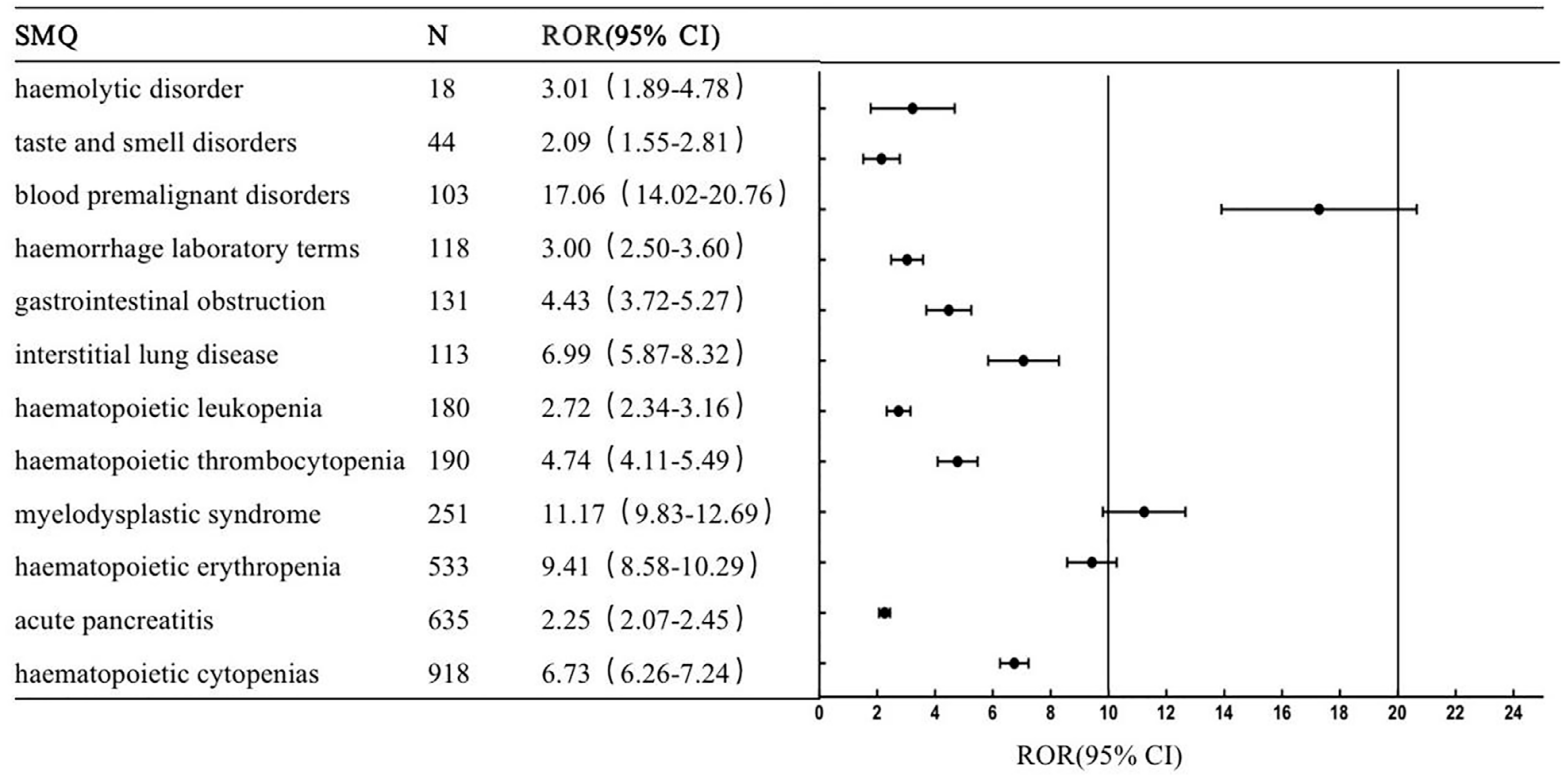
The positive signal distribution of Olaparib using standardized MedDRA queries.

**FIGURE 2:**
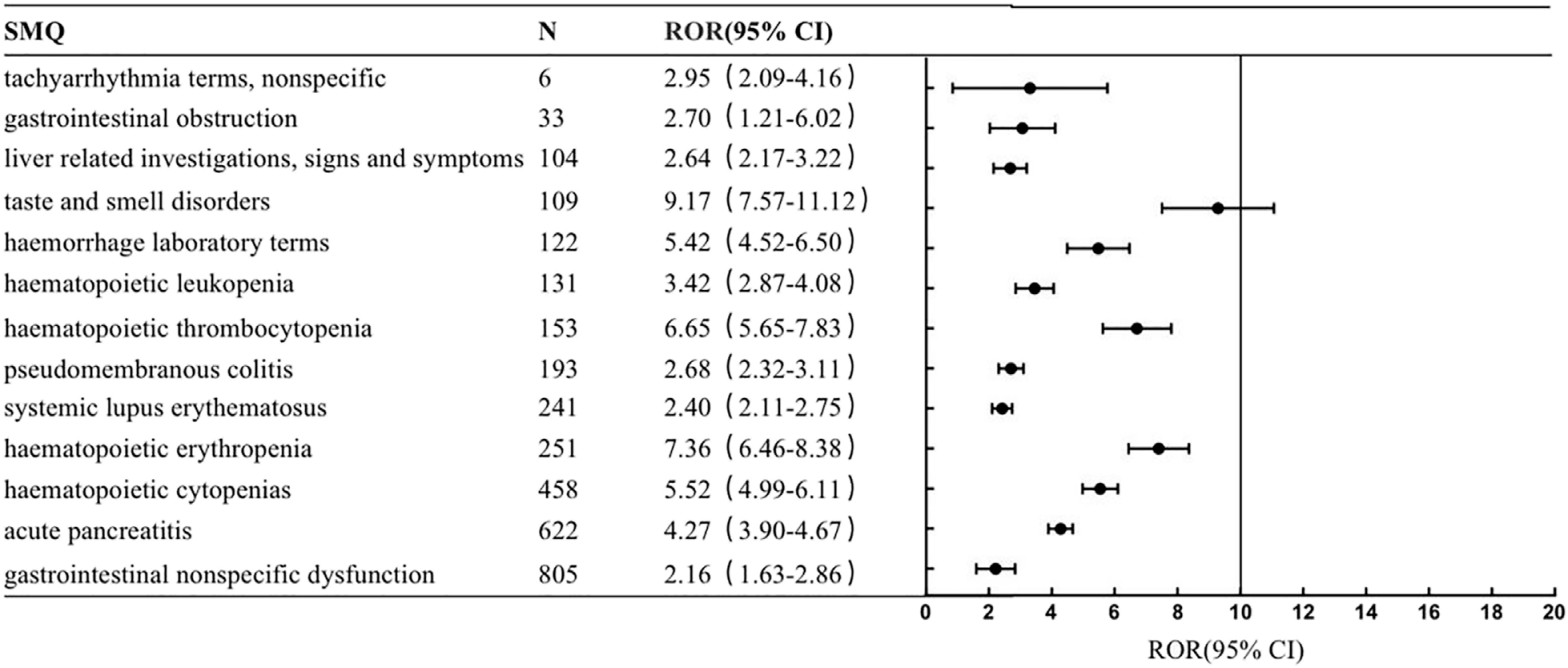
The positive signal distribution of Rucaparib using standardized MedDRA queries.

**FIGURE 3:**
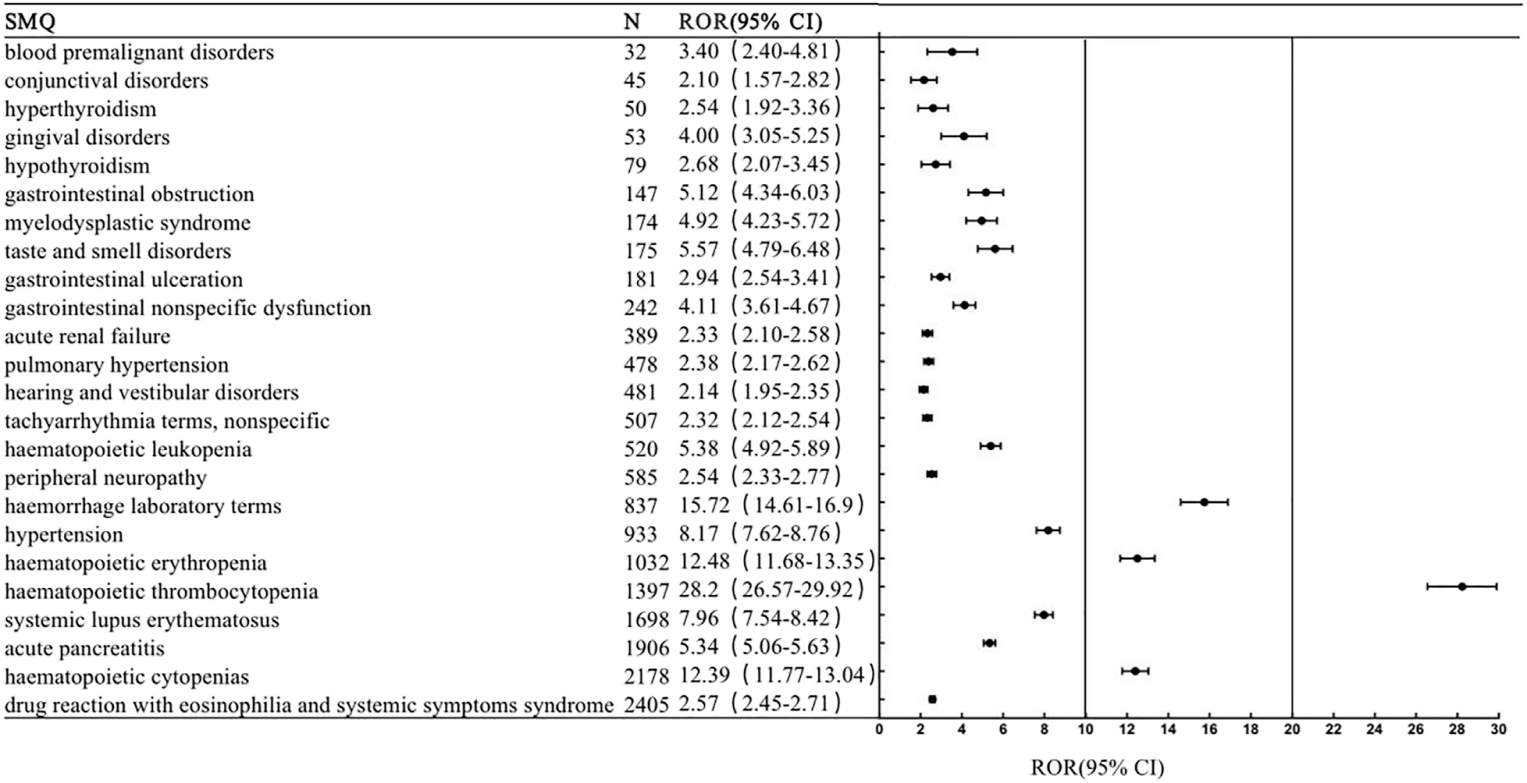
The positive signal distribution of Niraparib using standardized MedDRA queries.

**FIGURE 4:**
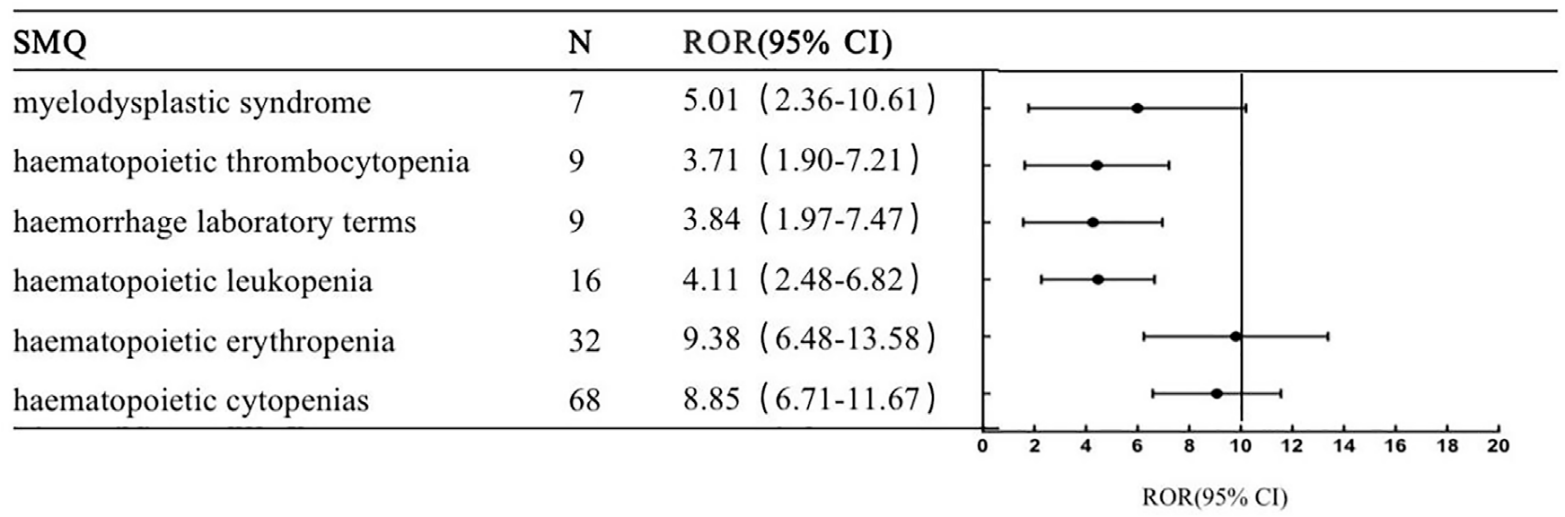
The positive signal distribution of Talazoparib using standardized MedDRA queriesable.

This study conducted a generalized SMQ search for PARPi and proceeded with signal detection to comprehensively discover specific clinical cases related to AEs reported for the four drugs. The results showed that olaparib involved a total of 12 positive signals, of which blood premalignant disorders had the strongest signal (ROR = 17.06), followed by myelodysplastic syndrome (ROR = 11.17) and hematopoietic erythropenia (ROR = 9.41). Other serious AEs included interstitial lung disease (ROR = 6.99), hematopoietic cytopenias (ROR = 6.73), hematopoietic thrombocytopenia (ROR = 4.74) and gastrointestinal obstruction (ROR = 4.43) (Figure 1). Rucaparib involved 13 positive signals, of which taste and smell disorders had the strongest signal (ROR = 9.17), followed by hematopoietic erythropenia (ROR = 7.36) and hematopoietic thrombocytopenia (ROR = 6.65) (Figure 2). Niraparib involved 24 positive signals, 4 of which had ROR values greater than 10, including hematopoietic thrombocytopenia (ROR = 28.2), hemorrhage laboratory terms (ROR = 15.72), hematopoietic erythropenia (ROR = 12.48) and hematopoietic cytopenia (ROR = 12.39) (Figure 3). In addition, other rare signals included hyperthyroidism, hypothyroidism, pulmonary hypertension, nonspecific tachyarrhythmia, hypertension, systemic syndrome with eosinophilia, etc. Talazoparib involved 6 positive signals, all of which were hematological system disorders, including hematopoietic erythropenia (ROR = 9.38), hematopoietic cytopenia (ROR = 8.85) and myelodysplastic syndrome (ROR = 5.01) (Figure 4).

### Signal of Preferred Terms

We further examined PT signals, which revealed both high RORs for niraparib and olaparib in blood and lymphatic system disorders and gastrointestinal disorders. The ROR was greater for niraparib than olaparib in terms of red blood cell count decrease (ROR = 70.47), platelet count decrease (ROR = 52.78), constipation (ROR = 29.77), hemoglobin decrease(ROR = 23.66), blood pressure fluctuation (ROR = 20.54) and lymphangioleiomyomatosis (ROR = 471.20). However, a high ROR was revealed for olaparib in the following PTs: myelodysplastic syndrome (ROR = 35.47), acute myeloid leukemia (ROR = 25.14) and ileus (ROR = 22.12) (Table 4). The RORs of AEs for rucaparib and talazoparib were relatively low. PTs with an ROR greater than 20 for rucaparib included renal impairment (ROR = 33.32), dysgeusia (ROR = 27.06)and photosensitivity reactions (ROR = 22.12). PTs with an ROR greater than 20 for talazoparib included pancytopenia (ROR = 25.81) and anemia(ROR = 20.82) (Table 4).

**TABLE 4:** Signal strength for PARPi agents based on PT level in FAERS.

SOC system		Olaparib		Rucaparib		Niraparib		Talazoparib	
		N	ROR(95% CI)	N	ROR(95% CI)	N	ROR(95% CI)	N	ROR(95% CI)
Gastrointestinal disorders	nausea	511	3.56(3.24–3.90)	481	6.23(5.64–6.88)	1836	10.30(9.76–10.87)		-
	vomiting	207	2.28(1.98–2.62(	157	3.00(2.55–3.53)	658	5.05(4.66–5.47)		-
	diarrhoea	130	1.16(0.98–1.38(	185	2.96(2.55–3.43)	354	2.13(1.91–2.17)		-
	constipation	75	2.07(1.65–2.60(	176	8.79(7.54–10.24)	1,316	29.77(28.01–31.63)		-
	ileus	55	22.12(16.93–28.90)	16	4.23(2.59–6.92)		-		-
	abdominal distension	39	2.05(1.49–2.81(	35	3.16(2.26–4.41)		-		-
	colitis	16	2.24(1.37–3.66)		-		-		-
	dyspepsia		-	30	2.59(1.81–3.71)		-		-
Blood and lymphatic system disorders	anaemia	473	14.10(12.82–15.52)	147	7.07(5.98–8.35)	392	7.27(6.57–8.06)	40	20.82(14.87–29.16)
	platelet count decreased	114	6.04(5.02–7.28(	106	9.77(8.04–11.87)	1,232	52.78(49.58–65.19)	9	8.01(4.12–15.57)
	myelodysplastic syndrome	113	35.47(29.38–42.82)		-	20	3.98(2.56–6.18)		-
	thrombocytopenia	96	4.68(3.83–5.75)	50	4.17(3.31–5.12)	333	11.08(9.92–12.38)	18	15.39(9.54–24.84)
	haemoglobin decreased	85	4.39(3.54–5.44)	77	6.88(5.48–8.63)	634	23.66(21.79–25.69)	9	7.86(4.04–15.29)
	bone marrow failure	83	15.94(12.82–19.82)		-		-		-
	acute myeloid leukaemia	80	25.14(20.13–31.40)		-	26	5.26(3.57–7.73)		-
	pancytopenia	71	7.04(5.56–8.90)		-		-	16	25.81(15.32–43.48)
	white blood cell count decreased	62	3.16(2.36–4.07)	50	4.39(3.32–5.81)	441	15.76(14.31–17.37)		-
	neutrophil count decreased	55	8.49(6.50–11.08)	12	3.13(1.78–5.53)	83	8.44(6.79–10.48)		-
	red blood cell count decreased	37	7.74(5.60–10.70)	44	15.90(11.79–21.43)	461	70.47(63.98–77.62)		-
	febrile neutropenia	20	2.03(1.31–3.15)	19	3.31(2.11–5.20)		-	4	6.85(2.55–18.39)
	leukaemia	15	7.84(4.72–13.03)		-		-		-
	lymphocyte count decreased	10	2.69(1.44–5.01)		-		-		-
	acute leukaemia	4	8.67(3.25–23.16)		-		-		-
	neutropenia		-	41	3.33(2.45–4.54)		-	17	14.16(8.66–23.15)
Cardiac and Vascular disorders	vasculitis	10	4.08(2.19–7.60)		-		-		-
	cardiac discomfort	4	12.37(4.63–33.07)		-		-		-
	atrial tachycardia	3	7.18(2.31–22.33)		-		-		-
	sinus disorder		-	11	4.93(2.73–8.91)	26	4.48(3.05–6.59)		-
	extrasystoles		-	4	4.06(1.52–10.84)		-		-
	blood pressure increased		-		-	696	17.81(16.46–19.28)		-
	heart rate increased		-		-	365	13.15(11.83–14.62)		-
	hypertension		-		-	343	5.49(4.93–6.13)		-
	palpitations		-		-	183	4.87(4.20–5.64)		-
	blood pressure fluctuation		-		-	90	20.54(16.66–25.33)		-
	thrombosis		-		-	74	2.60(2.07–3.27)		-
	supraventricular tachycardia		-		-	26	4.48(3.05–6.59)		-
	lymphangioleiomyomatosis		-		-	16	471.20(267.46–830.15)		-
	hypertensive crisis		-		-	11	3.02(1.76–5.47)		-
	heart rate abnormal		-		-	10	6.95(3.73–12.95)		-
Skin and subcutaneous tissue disorders	stomatitis	29	2.83(1.96–4.07(	28	4.69(3.23–6.81)		-		-
	erythema nodosum	7	10.33(4.91–21.72)		-		-		-
	dermatomyositis	4	8.42(3.15–22.48)		-		-		-
	skin erosion	3	6.08(1.96–18.90)		-		-		-
	photosensitivity reaction		-	44	21.77(16.15–29.36)	99	18.92(15.51–21.10)		-
	dry skin		-	22	3.41(2.24–5.19)	61	3.64(2.83–4.89)		-
	sunburn		-	12	15.64(8.86–27.61)		-		-
	petechiae		-	5	4.14(1.72–9.95)	61	19.79(15.35–25.50)		-
	lip blister		-	4	12.54(4.70–33.50)		-		-
	noninfective gingivitis		-	3	6.08(1.96–18.90)		-		-
	skin atrophy		-	3	5.38(1.73–16.71)		-		-
	hot flush		-		-	127	6.65(5.57–7.92)		-
	skin discolouration		-		-	55	4.44(3.40–5.49)		-
	mucosal inflammation		-		-	16	2.26(1.38–3.70)		-
Hepatobiliary disorders	hepatic failure	16	2.11(1.36–3.61)		-		-		-
	hepatic enzyme increased		-	31	4.32(3.03–6.15)	41	2.18(1.60–2.97)		-
Renal and urinary disorders	renal impairment	65	3.92(3.96–5.00)	14	33.32(19.66–54.67)	126	5.02(4.21–5.99)		-
	blood creatinine increased	58	4.35(3.36–5.64)		-	234	11.88(10.42–13.54)		-
	renal failure	45	2.45(1.63–5.31)		-		-		-
	glomerular filtration rate decreased	10	5.43(2.92–10.11)	5	4.64(1.93–11.16)	36	12.89(9.34–18.04)		-
	urinary tract infection		-	41	2.37(1.74–3.23)	192	4.34(3.76–5.01)		-
Musculoskeletal and connective tissue disorders	back pain		-		-	285	4.23(3.76–4.77)		-
	myalgia		-		-	148	2.65(2.25–3.12)		-
	muscle spasms		-		-	146	2.62(2.22–3.08)		-
	muscular weakness		-		-	72	2.02(1.61–2.56)		-
	musculoskeletal pain		-		-	55	2.85(2.19–3.72)		-
	osteopenia		-		-	22	5.15(3.38–7.82)		-
	pain in jaw		-		-	20	2.09(1.35–3.25)		-
Respiratory, thoracic and mediastinal disorders	interstitial lung disease	100	11.31(7.27–13.79)		-		-		-
	pneumonitis	45	10.64(7.92–14.28)		-		-		-
	pneumocystis jirovecii pneumonia	17	13.46(8.35–21.71)		-		-		-
	dyspnoea		-	10	2.87(1.54–5.34)	446	2.76(2.51–3.04)		-
	cough		-		-	277	4.00(3.54–4.51)		-
	upper-airway cough syndrome		-		-	26	13.19(9.30–20.16)		-
	pulmonary mass		-		-	24	8.39(5.62–12.55)		-
Other	fatigue	362	2.71(2.44–3.02)	545	8.15(7.41–8.96)	1703	10.40(9.84–10.99)		-
	decreased appetite	129	3.45(2.89–4.11)	112	5.20(4.30–6.28)	641	12.17(11.22–13.21)		-
	neuropathy peripheral	59	3.79(2.93–4.91)	32	3.51(2.48–4.98)	362	16.11(14.49–17.92)		-
	dysgeusia	39	2.42(1.77–3.32)	16	27.06(16.52–44.31)	137	5.67(4.79–6.72)		-
	initial insomnia		-	4	3.59(1.34–9.57)	851	10.83(10.08–11.63)		-
	thyroid function test abnormal		-	3	5.63(1.81–11.48)		-		-
	memory impairment		-		-	131	3.04(2.55–3.61)		-
	balance disorder		-		-	125	4.56(3.82–5.44)		-
	performance status decreased		-		-	49	32.18(24.22–42.75)		-
	haematochezia		-		-	34	2.74(1.96–3.84)		-
	epistaxis		-		-	81	3.47(2.78–4.32)		-
	gingival bleeding		-		-	28	6.71(4.62–9.73)		-

ROR, 1-5 ROR, 5–10 ROR, 10–15 ROR, 15–20 ROR >20 “-”

## Discussion

This large real-world comparison of PARPis therapy leveraged FAERS data and demonstrated key differences between the four FDA-approved PARPis in the risk of AEs, likelihood of gastrointestinal disorders, blood system disorders, cardiac disorders, etc. Overall, three main findings emerged: 1) Many organs or tissues can be involved, although some AEs occur much more commonly than others. We found SDR for the hematological system, gastrointestinal tract, cardiovascular system, liver, skin, lung and nervous system. However, we did not find SDR for endocrine systems, such as thyroid dysfunction, hypophysitis, and adrenal insufficiency; 2) SDR in FAERS showed a higher strength in blood system disorders and gastrointestinal disorders for all four PARPis; 3) SDR for serious AEs and rare AEs were significantly different among the four PARPi. A higher prevalence of constipation, decreased platelet count and decreased red blood cell count was found with niraparib vs. olaparib, and a higher prevalence of myelodysplastic syndrome and acute myeloid leukemia was found with olaparib vs. niraparib. The PARP inhibitor most associated with cardiovascular toxicity is niraparib. Rucaparib and niraparib had the higher incidence of photosensitivity reaction. Olaparib showed strong signals of interstitial lung disease, etc.

From a pharmacological point of view, PARPis share similar mode of action but are different in many characteristics. Table 5 lists the differences in pharmacokinetics of the four PARPis (D. Sandhu et al., 2022; Valabrega et al., 2021). Regarding pharmacodynamic, all PARPis are able to bind to the NAD + binding site through a benzamide core pharmacophore. However, PARPis have different binding affinity for the different PARP family members, as reported in Table 5 (Thorsell et al., 2017). In addition, an off-target activity has been described for PARPis, and relevant differences about polypharmacology have been found for the different molecules. The above differences in pharmacology may lead to differences in AEs in clinical practice.

**TABLE 5:** Pharmacological properties of the four PARPis.

	Olaparib	Rucaparib	Niraparib	Talazoparib
Pharmacokinetics				
Bioavailability	NA	36%	73%	41%
Volume of distribution (L)	(T) 158 (C) 167	420	1,074	420
Half-life (h)	(T) 15 (C) 11.9	25.9	36	90
Tmax (h)	(T) 1.5 (C) 1–3	1.9	3	1–2
Cmax (nM)	13,400	6,000	2,500	43
metabolism	CYP3A4/5	CYP2D6	carboxylesterases	glucuronide conjugation
Renal excretion	44%	17.4%	47.5%	68%
Clearance (L/h)	(T) 7 (C) 8.6	6.5	16.2	6.5
PPB	82%	70.2%	83%	74%
Posology	BID (300 mg)	BID (600 mg)	OD (300 mg)	OD (1 mg)
On-target efficiency (median IC50 values in nM)				
PARP1	13	80	35	3
PARP2	56	83	8	4
PARP3	99	460	380	63
PARP4	409	835	408	254
Off-target efficiency (median IC50 values in nM)				
DYRK1A	--	NA	297	--
DYRK1B	--	747	254	--
CDK16	--	381	--	--
PIM3	--	436	NA	--
DAT	NA	NA	51	NA
NET	NA	NA	239	NA
SERT	NA	NA	363	NA
OCT1	--	4,300	NA	NA
MATE1	5,500	630	--	NA
MATE2-K	--	190	NA	NA
5-HT4	NA	∼1,000	∼1,000	NA
hERG	NA	--	∼1,000	NA

Abbreviatons: “-”, no inhibition; NA, not available; (T), tablet formulation; (C), capsule formulation; PPB, plasma protein binding; Cmax, peak serum concentration; Tmax, time taken to reach Cmax; IC50, half maximum inhibitory concentration.

### Gastrointestinal Disorders

Gastrointestinal toxicities are mediated via off-target kinase inhibition, These types of AEs (e.g., vomiting) are common for kinase inhibitors. In our study, gastrointestinal disorders were common with treatment with olaparib, niraparib and rucaparib but rare with talazoparib. We found that the overall signals for niraparib and rucaparib were stronger than those for olaparib, including symptoms of nausea, vomiting, diarrhea, and constipation, but olaparib had a strong signal in the ileus at the PT level. This was consistent with several systematic reviews (Bao et al., 2021). Gastrointestinal toxicity is one of the most frequent AEs associated with PARPis, often occurring early after treatment initiation. However, approximately 70% of patients have mild AEs, and only 3–4% of patients will have severe (grade ≥3) AEs associated with PARPis (Poveda et al., 2021; Wu et al., 2021). In addition, a higher ROR for olaparib was found in ileus (ROR = 22.12) compared to other drugs, which has not been reported in previous studies. Further observation and research are needed. PARPis have different pharmacological properties. Generally, PARPis can be taken on an empty stomach or with meals. Niraparib is metabolized by carboxylesterase, but other PARPis are metabolized by the cytochrome P450 enzymatic pathway (CYP) (Ledermann et al., 2016; Lee, 2021; H.; Wang et al., 2020). Clinically, for patients with severe nausea and vomiting, it is recommended to give prokinetics or antihistamines 60 min before taking PARPi for prevention but to avoid the use of NK-1 inhibitors such as aprepitant, which is a strong CYP3A4 inhibitor and affects the metabolism of olaparib (LaFargue et al., 2019).

### Hematologic Toxicity

Hematologic toxicities are a common class of effects of PARPis; they are also the most common cause of dose modification, interruption, and discontinuation. In our study, the ROR signals were higher with niraparib, followed by olaparib and then rucaparib. Red blood cell count and platelet count decreased. Nirapalib is the only PARPi that breaks through the limitations of BRCA and expands the front-line maintenance treatment of ovarian cancer to the entire population (Gonzalez-Martin et al., 2019). A key feature of niraparib is its selectivity for PARP-1/2, which is 100 times higher than that of other PARP enzymes. Compared with other PARPis, niraparib has stronger PARP capture activity, which is conducive to its good antitumor efficacy in BRCAwt patients (S. K. Sandhu et al., 2013; D. Wang et al., 2018). A previous study showed that inhibition of PARP-2 affected the differentiation of erythroid cells and reduced the lifespan of red blood cells, leading to red blood cell reduction and anemia (Farres et al., 2015). In addition, exposure to bone marrow is determined by the volume of distribution (Vd) of a drug. with a higher Vd leading to increased distribution into bone marrow (Diaz et al., 2013). Niraparib has a Vd of 1074 L, significantly higher than others (Table 5), so would be expected to have a higher number of cases. After 5-6 cycles of niraparib administration, the incidence of anemia (≥ grade 3) was 25% in one RCT (Mirza et al., 2016). Anemia causes a decline in physical performance, and as expected, a high ROR of performance status decrease was also detected for niraparib in our study. On the other hand, we found that the ROR value of the thrombocytoplasmic signal was the highest in the SMQs for niraparib, consistent with the RCT data. Niraparib induced thrombocytopenia in a dose-dependent manner. A subsequent *post hoc* analysis reported that patients with a weight of less than 77 kg or a baseline platelet count of less than 150,000/mm^3^ benefited from a reduced starting dose of 200 mg once daily (Berek et al., 2018) in the ENGOT-OV16/NOVA study. In a retrospective cohort study of US health care claims data, fewer patients receiving niraparib (69.3%) than olaparib (89.4%) or rucaparib (93.2%) started treatment at the highest indicated dose (Arend et al., 2021). The above results suggest that modified niraparib doses were implemented in clinical practice to mitigate the risk of severe hematologic toxicity.

In our study, rare and severe hematological toxicities of olaparib were significantly stronger than those of niraparib and rucaparib, including MDS (N = 113; ROR = 35.47), bone marrow failure (N = 83; ROR = 15.94), and AML (N = 80; ROR = 25.14), which are worthy of vigilance in clinical medication. Bolton et al. found that compared with traditional chemotherapeutics, PARPis could cause acquired mutations of clonal hematopoiesis in the blood system through DNA damage, thereby increasing the risk of secondary MDS and AML. A meta-analysis suggested that compared with placebo, PARPi significantly increased the risk of MDS and AML, with an incubation period of approximately 17.8 months (Morice et al., 2021). The risk associated with nirapali is higher than that with olaparib (OR = 2.58 vs. OR = 1.45), which is consistent with the results of this study. In addition, Platinum and taxane-based chemotherapy is one of the standard first-line chemotherapy regimens for ovarian, table3 showed that top three concomitant medications for olaparib are carboplatin, bevacizumab, paclitaxel. Superimposed myelosuppression induced by platinum and paclitaxel may also be responsible for severe hematological toxicities. Real-world pharmacovigilance data suggest that anemia is most relevant to the diagnosis of MDS and AML. Patients with anemia should be more alert to the occurrence of MDS and AML. In addition, the data on talazoparib only involved the blood system, such as anemia and thrombocytopenia, but the data were limited.

### Cardiovascular Toxicity

A large variety of cardiotoxicity events, with manifestations such as increased blood pressure (N = 696; ROR = 17.81), increased heart rate (N = 365; ROR = 13.15), hypertension (N = 343; ROR = 5.49) and lymphangioleiomyomatosis (N = 16; ROR = 471.20), were reported in niraparib. In the NOVA trial, only 9% of patients treated with this product developed III-IV hypertension, with a median duration of 15 days (Del Campo et al., 2019). However, a single-center retrospective study found (reported by the 2021 ESMO Conference) that almost half of patients taking PARPi experienced a cardiovascular event. PARP inhibitors at lower drug concentrations may cause inhibition of functional proteins outside the PARP target (off-target effects), leading to corresponding AEs. Niraparib has relatively obvious off-target effects and has a strong effect on targets such as serotonin transporter (SERT), dopamine transporter (DAT), and norepinephrine transporter (NET), which may be related to adverse reactions such as high blood pressure (Montastruc et al., 2020; Staropoli et al., 2018). The unique inhibition of DYRK1A by niraparib could also contribute to the hypertension reported (Table 5). As niraparib inhibits DYRK1A, increased levels of these neurotransmitters would be seen,which in turn have inotropic effects on the heart, causing high blood pressure (London et al., 2018). Inhibition of the Kv11.1 (hERG) potassium ion channel causes QT prolongation resulting in arrhythmia (Sanguinetti and Tristani-Firouzi, 2006). Niraparib is weak known features of potential hERG inhibitors, which may explain why arrhythmia cases are, so far, unique to niraparib. Prospective research and long-term follow-up of cardiovascular diseases are necessary to grasp the balance between the efficacy and safety of PARPis correctly, deal with AEs in a timely and effective manner, and help patients maintain long-term medication. Before prescription, baseline measurement of cardiovascular-related indicators should be carried out, and the underlying cardiovascular diseases should be actively dealt with to maximize the benefits of patients. Furthermore, there were 16 cases of lymphangioleiomyomatosis with niraparib in this study. It is a rare disease that mainly affects the lungs. The typical manifestation is diffuse cystic changes. Studies have shown that its onset has a certain relationship with estrogen (LaFargue et al., 2019), therefore, lymphangioleiomyomatosis may be related to ovarian cancer itself. Whether it is related to PARPis needs further study.

### Skin and Musculoskeletal Toxicity

Skin and subcutaneous tissue disorders from PARPis therapy are rare but clinically important.

The most commonly reported dermatological AEs with niraparib and rucaparib were photosensitivity reactions and dry skin, and stomatitis was the most commonly reported with olaparib in our study. We found that olaparib had lower dermatological AEs. In addition, 61 patients had subcutaneous petechia with niraparib, which may be related to subcutaneous hemorrhage caused by niraparib-induced thrombocytopenia. Patients should pay attention to Sun protection and moisturize their skin during treatment with PARPis medication.

In previous studies, back pain and joint pain occurred occasionally, and the overall incidence was less than 35%, of which incidence of grade ≥3 was less than 0.5% (LaFargue et al., 2019) he overall incidence of back pain with olaparib was 11.3%, and the incidence of arthralgia was 14.9%, while the overall incidence of back pain was 13.4% in the population with a fixed starting dose of niraparib (300 mg, once a day) and was only 7.9% in the individualized initial dose population (200 or 300 mg, once a day) (Mirza et al., 2020; Bao et al., 2021). We found a similar disproportionality trend for musculoskeletal toxicity with niraparib but no reports with other PARPis.

### Others

We found that a small number of cases of liver and kidney damage were reported in FAERS, both for olaparib, niraparib and rucaparib, which mainly manifested as increased liver enzymes and serum creatinine. Rucaparib inhibits kidney transporter proteins multidrug and toxin extrusion (MATE1) and MATE2 (multidrug and toxin extrusion protein 2, MATE2-K) (Liao et al., 2020), which affect the secretion of creatinine, whilst niraparib does not inhibit MATE1 and is not related to elevated serum creatinine. Olaparib alsoinhibits MATE1 with IC50 < 10 μM and is also associated with elevated creatinine levels (McCormick and Swaisland, 2017).

It should be noted that ROR indicated an increased risk of serious respiratory AEs for olaparib, including interstitial lung disease pneumonitis and Pneumocystis jirovecii pneumonia. A meta-analysis compared PARPi-induced pneumonia (Ma et al., 2021), showing that the incidence of pneumonia was 0.79%, and the risk signal of olaparib was 11.44, similar to the results of this study. We found that niraparib had relatively mild respiratory-related AEs, including upper airway cough syndrome and dyspnea.

A wide array of neurologic AEs were associated with niraparib treatment, including initial insomnia, memory impairment, neuropathy peripheral dysgeusia and balance disorder. The ROR signals suggested that neurotoxicity might be more frequent in patients treated with niraparib than olaparib. Niraparib is a relatively strong DAT inhibitor with IC50 value of 51 nM (Table 5); meanwhile DAT as a dopamine transporter has a clear role in schizophrenia and deliria (Hahn and Blakely, 2002), therefore it is common neurologic disorder observed with niraparib.

### Limitations

The FAERS database is a spontaneous reporting database; as such, there may be underreporting. At the same time, most PARPis are oral maintenance medications taken outside of the hospital; therefore, patients’ spontaneous reports account for a certain proportion. The data may not be complete enough, so it is impossible to accurately analyze related factors, such as drug dosage and treatment course. In addition, all signal detection results can only suggest that there is a statistical correlation; whether there is a real causal relationship still needs further evaluation and research.

## Conclusion

This study confirms that the occurrence of AEs associated with niraparib is higher than that associated with olaparib and is related to the gastrointestinal, hematological, cardiovascular and neurologic systems. In addition, this study also reveals that olapali has serious and rare AEs, such as AML, MDS, ileus and interstitial lung disease. Our findings based on FAERS data showed real-world trends of reported PARP signals and potential rank-order in terms of susceptibility, which are in agreement with the results from previous studies. This suggests the usefulness of the FAERS database and the data mining method used herein, and it will benefit clinical management pharmacovigilance research to potentially improve treatment safety with PARPis.

## Data Availability

The original contributions presented in the study are included in the article/Supplementary Material, further inquiries can be directed to the corresponding author.
